# Immune activation and inflammatory biomarkers as predictors of venous thromboembolism in lymphoma patients

**DOI:** 10.1186/s12959-022-00381-3

**Published:** 2022-04-19

**Authors:** Vladimir Otasevic, Biljana Mihaljevic, Natasa Milic, Dejana Stanisavljevic, Vojin Vukovic, Kristina Tomic, Jawed Fareed, Darko Antic

**Affiliations:** 1 Lymphoma Center, Clinic for Hematology, University Clinical Center of Serbia, Belgrade, Serbia; 2 Faculty of Medicine, University of Belgrade, Belgrade, Serbia; 3 Institute for Medical Statistics and Informatics, Faculty of Medicine, University of Belgrade, Belgrade, Serbia; 4 Loyola University Medical Center, Maywood, USA

**Keywords:** Inflammation, Lymphoma, Risk factor, Venous thromboembolism, Anticoagulants, Biomarkers, C-reactive protein, Endothelial cells, Hematologic neoplasms, Immunity

## Abstract

**Background:**

Lymphomas are characterized by elevated synthesis of inflammatory soluble mediators that could trigger the development of venous thromboembolism (VTE). However, data on the relationship between specific immune dysregulation and VTE occurrence in patients with lymphoma are scarce. Therefore, this study aimed to assess the association between inflammatory markers and the risk of VTE development in patients with lymphoma.

**Methods:**

The erythrocyte sedimentation rate (ESR), C-reactive protein (CRP), neutrophil-to-lymphocyte ratio (NLR), platelet-to-lymphocyte ratio (PLR), lactate dehydrogenase (LDH), total protein (TP), and albumin were assessed in 706 patients with newly diagnosed or relapsed lymphoma. Data were collected for all VTE events, while the diagnosis of VTE was established objectively based on radiographic studies. ROC (receiver operating characteristic) curve analysis was performed to define the optimal cutoff values for predicting VTE.

**Results:**

The majority of patients was diagnosed with aggressive non-Hodgkin lymphoma (58.8%) and had advanced stage disease (59.9%). Sixty-nine patients (9.8%) developed VTE. The NLR, PLR, ESR, CRP, and LDH were significantly higher in the patients with lymphoma with VTE, whereas the TP and albumin were significantly lower in those patients. Using the univariate regression analysis, the NLR, PLR, TP, albumin, LDH, and CRP were prognostic factors for VTE development. In the multivariate regression model, the NLR and CRP were independent prognostic factors for VTE development. ROC curve analysis demonstrated acceptable specificity and sensitivity of the parameters: NLR, PLR, and CRP for predicting VTE.

**Conclusion:**

Inflammatory dysregulation plays an important role in VTE development in patients with lymphoma. Widely accessible, simple inflammatory parameters can classify patients with lymphoma at risk of VTE development.

**Supplementary Information:**

The online version contains supplementary material available at 10.1186/s12959-022-00381-3.

## Introduction

Venous thromboembolism (VTE) is a leading cause of cancer-associated thrombosis (CAT) in patients with malignancy. The pathophysiological relationship between VTE development and malignancy was established decades ago [1]. Patients with cancer have up to 7-times higher risk of developing VTE than do healthy individuals [2]. Moreover, VTE is the second leading cause of mortality in patients with cancer immediately after cancer progression [3]. Additionally, VTE prolongs the duration of hospitalization and consequently raises the costs of treatment [4]. Furthermore, Khorana et al. [3] identified CAT as the leading cause of mortality in ambulatory patients with active malignancy receiving chemotherapy. The clinical consequences and financial burden of VTE in specific groups of cancer patients have been gaining increased attention [5].

Lymphomas comprises a heterogenous group of clonal hematological neoplasms that are characterized by varying clinical course, from utterly indolent to extremely aggressive [6]. Their biological diversity is reflected in various mechanisms through which the disease advances and causes complications. A particular pathophysiological feature of lymphomas is immune dysregulation, which is deeply related to activation of inflammation. Multiple pro-inflammatory cytokines have a principal role in lymphomagenesis: interleukin (IL) 6 (IL-6) is related to Th17 immune response that has association with non-Hodgkin lymphoma (NHL). In addition, IL-10 gene polymorphisms are linked to elevated risk of NHL development and higher levels of tumor necrosis factor alpha (TNF-α) are associated with developing particular types of NHL [7]. The level and nature of inflammation dysregulation vary between different types of lymphomas [8]. In patients with a compromised immune system due to both malignancy (primary disease) and cancer treatment, the possibility of infections is substantial, which can generate circumstances for VTE complications [9]. The pathways that trigger inflammation are subject to fine modulation and differ based on the type of lymphoma. However, inflammation has been classically assessed by white blood cell (WBC) count and standardized acute phase reactants including C-reactive protein (CRP), sedimentation rate, and fibrinogen level. Moreover, the neutrophil-to-lymphocyte ratio (NLR) and platelet-to-lymphocyte ratio (PLR) are newer biomarkers for systemic inflammation [10]. The NLR and PLR have been widely studied in different medical fields, where they have demonstrated prognostic significance of various outcomes [11–14]. Additionally, their potential strength for predicting VTE in cancer patients receiving chemotherapy has been emphasized in several recently published studies [15, 16]. However, their predictive performance and reliability have not been evaluated in patients with lymphoma.

Inflammation and inflammation-related conditions are associated with an increased risk of VTE due to dysregulation of multiple pathophysiological pathways, including venous stasis, hypercoagulability, inflammation, IL-6 expression, and inhibition of natural inhibitors of coagulation and anticoagulants [17]. The inflammatory and thrombotic pathways overlap [17]. Regarding responses to inflammation, besides other stimuli, endothelial cells transform towards prothrombotic phenotype by increasing expression of leukocyte adhesion molecules (P- and E-selectin), tissue factor (TF), and angiopoietin2 (Angpt2). These modifications result in a loss of vascular integrity coupled with lowered expression of antithrombotic molecules and overexpression of procoagulants (complement effectors, coagulation factors, thrombin, and VIIa and Xa) [18]. Furthermore, inflammatory cytokines, such as P-selectin, formation of luminal von Willebrand factor, and TF expression lead to recruitment and activation of monocytes, neutrophils, platelets, and coagulation activation [18, 19]. At the same time, fibrinolysis system is partially impaired by elevated levels of prothrombin activator inhibitor-1 (PAI-1) [18]. Immunothrombosis, a relatively new term coined by Engelmann and Massberg, [20] emphasizes the role of innate immunity in VTE development. However, data on the relationship between specific immune dysregulation in patients with lymphoma and VTE occurrence are scarce. Therefore, our study aimed to assess the association between inflammatory markers and the risk of VTE development in patients with lymphoma receiving chemotherapy and evaluate the relationship between VTE and treatment course in patients with lymphoma.

## Methods

### Study population

The study included 706 patients with newly diagnosed or relapsed lymphomas (including NHL and Hodgkin lymphoma [HL] and excluding chronic lymphocytic leukemia [CLL] and the leukemic phases of all lymphomas) at the Clinic for Hematology, University Clinical Center of Serbia (UCCS). The study protocol has been approved by the UCCS’s Ethics Committee, and written informed consent was obtained from all participants. The time frame in which eligible patients were recruited was from January 2010 to November 2019. Patients with CLL and other leukemic phases of lymphomas were excluded because their differential WBC count is shifted and affect the NLR and PLR, rendering these values invalid. Data of patients with newly diagnosed and relapsed lymphoma were collected for all VTE events. VTE was diagnosed objectively based on radiographic studies, including compression ultrasonography, contrast-enhanced thoracic computed tomography, and magnetic resonance imaging of central nervous system (CNS) thrombosis, as well as clinical examination and laboratory evaluation. All probable cases of VTE were reviewed by a final diagnosis committee composed of two specialists (an internist and a radiologist).

### Laboratory investigations

Blood samples from patients with lymphoma included in the study were collected using vacuum tubes. Samples were anticoagulated with EDTA, and machine-automatized complete blood count (CBC) with leukocyte differential counts was performed. The NLR and PLR were calculated using the CBC with leukocyte differential counts. Citrated blood samples were analyzed in batches using commercially available ELISA kits at the University Clinical Center of Serbia. Furthermore, the following biochemical parameters were analyzed: erythrocyte sedimentation rate (ESR), C-reactive protein (CRP), lactate dehydrogenase (LDH), fibrinogen, total protein (TP), and albumin.

### Statistical analysis

Categorical variables are displayed as counts with percentages, and numerical variables are presented as medians with ranges. Normality of distribution was assessed using the Kolmogorov–Smirnov test. LDH, TP, and albumin were transformed to dichotomous categorical variables and defined as “under the lower reference range limit,” “in the reference range,” or “over the upper reference range limit.” Differences between patients with lymphoma who developed thrombosis and those without thrombosis were assessed using the Mann-Whitney test for numerical variables and the chi-square test for categorical variables. ROC (receiver operating characteristic) curve analysis was used to define the best cutoff values for predicting VTE. Multivariate logistic regression analysis was performed to identify significant predictors of thrombosis in patients with lymphoma. Significant variables from the univariate logistic regression analysis were fitted into the multivariate analysis. The results are presented as odds ratios (ORs) with corresponding 95% confidence intervals (CIs). Statistical significance was set at *p* < 0.05. Statistical analysis was performed using IBM SPSS statistical software (SPSS for Windows, release 25.0, SPSS, Chicago, IL, USA).

## Results

The mean age of the patients included in the study was 52.8 years (range, 18–89 years); 53% of the patients were men. A total of 415 patients (58.8%) had aggressive NHL, 172 (24.3%) had indolent NHL, and 119 (16.9%) had HL. Most of the patients were newly diagnosed (90.4%) and had advanced stage disease, with Ann Arbor stages III and IV accounting for 20.6 and 39.3% of the cases, respectively. Most patients had good performance status (Eastern Cooperative Oncology Group Performance Status [ECOG PS] 0–1: 81.7%;), and B symptoms were present in 55.8% of patients. A “bulky” tumor mass (lymphoma masses or conglomerate of lymph node masses that measure ≥7 cm) was observed in 30.7% of the patients, and mediastinal involvement was found in 31.4% of the patients.

The median follow up was 25 months. Sixty-nine patients (9.8%) developed VTE events: 39 developed deep vein thrombosis (DVT) of the extremities, three developed abdominal vein thrombosis, 12 developed superficial vein thrombosis, 11 developed jugular vein thrombosis, and 16 developed pulmonary embolisms (some patients had more than one thrombotic event). Most patients (59.4%) had symptomatic VTE (41/69). The majority of patients developed VTE during treatment (52.1%). However, 46.5% of patients were diagnosed with VTE prior to treatment initiation, and 1.2% developed VTE after completion of treatment. VTE was more frequent in the patients with aggressive lymphoma (11.8%) than in those with HL (8.4%) and indolent lymphoma (5.3%) (Table 1). None of the patients with lymphoma with disease dissemination in the CNS developed VTE. Most patients with lymphoma with VTE had advanced stage disease (stage III, 29%; stage IV, 34.8%).

**Table 1:** VTE incidence according to type of lymphoma

Lymphoma type	VTE n (%)
Indolent NHL	9/170 (5.3%)
Aggressive NHL	49/415 (11.8%)
SLL	1/2 (50%)
Hodgkin lymphoma	10/119 (8.4%)

Legend: *VTE* venous thromboembolism, *NHL* non-Hodgkin lymphoma, *SLL* small lymphocytic lymphoma

The demographic and clinical characteristics of lymphoma patients with and without VTE are presented in Table 2. Compared with patients without VTE, the NLR, PLR, ESR, CRP, and LDH were significantly higher in the patients with lymphoma with VTE (*p* = 0.001, *p* = 0.001, *p* = 0.023, *p* < 0.001, and *p* = 0.035, respectively), whereas the TP and albumin were significantly lower (*p* = 0.024 and *p* = 0.032, respectively). In the patients with diffuse large B-cell lymphoma (DLBCL), IPI > 1 (intermediate/high risk group) was more frequent in those with VTE (*p* = 0.027).

**Table 2:** Demographic and clinical characteristics of lymphoma patients with and without VTE

Characteristic	Lymphoma patients without VTE	Lymphoma patients with VTE	p
Age, median (range)	56 (18–87)	54 (19–89)	0.898
Men/women	337/300	37/32	0.900
Hemoglobin, g/L, median (range)	124 (51–172)	117 (87–141)	0.017
Leukocytes, ×10^9^/L, median (range)	7.4 (0.4–28.5)	8.5 (4.6–16.6)	0.132
Platelets, ×10^9^/L, median (range)	248 (29–613)	283 (103–678)	0.034
NLR, median (range)	2.7 (0.2–32.5)	3.79 (0.7–160.5)	0.001
PLR, median (range)	10.1 (0.3–193.3)	14.5 (483.3)	0.001
ESR, mm/h, median (range)	26 (2–150)	38 (2–150)	0.023
CRP, mg/L, median (range)	9.9 (0.1–274.6)	30.6 (0.8–251.8)	< 0.001
Fibrinogen, mg/L, median (range)	5.3 (1–13.2)	5.7 (1.9–11.8)	0.351
LDH > reference range limit, n (%)	187 (30.4)	32 (50.8)	0.001
TP < reference range limit, n (%)	258 (40.5)	39 (56.6)	0.024
Albumin < reference range limit, n (%)	70 (11)	15 (21.2)	0.032
B symptomatology, n (%)	360 (56.6)	52 (75.4)	0.001
“Bulky” tumor mass, n (%)	182 (28.5)	34 (49.3)	< 0.001
Extranodal localization, n (%)	368 (57.8)	35 (50.7)	0.259
Mediastinal involvement, n (%)	189 (29.6)	32 (46.4)	0.004
IPI > 1, n (%)	153 (52.0)	27 (71.1)	0.027

Legend: *VTE* venous thromboembolism, *min* minimum, *max* maximum, *NLR* neutrophil to lymphocyte ratio, *PLR* platelet to lymphocyte ratio, *ESR* erythrocyte sedimentation rate, *CRP* C-reactive protein, *LDH* lactate dehydrogenase, *TP* total proteins, *IPI* international prognostic index (calculated for DLBCL)

In the univariate regression analysis, the NLR, PLR, TP, albumin, LDH, and CRP were found to be prognostic factors for VTE development in the patients with lymphoma (Table 3). In the subgroup analysis of superficial vein thrombosis events exclusion, both NLR and PLR remained prognostic factors for VTE development. B-symptomatology, a “bulky” tumor mass, mediastinal involvement, and ECOG PS were the significant clinicopathological prognostic factors for VTE development in the patients with lymphoma (*p* = 0.001, *p* = 0.001, *p* = 0.005, and *p* = 0.015, respectively) (Table 3). In the multivariate regression model, the NLR and CRP were found to be independent prognostic factors for VTE development in the patients with lymphoma (*p* = 0.046, OR = 1.043, 95% CI: 1.001–1.087 and *p* = 0.024, OR = 1.007, 95% CI: 1.001–1.013, respectively).

**Table 3:** Univariate regression models of TP, albumin, LDH, CRP, ESR, NLR, PLR, B-symptomatology, “bulky” tumor mass, mediastinal involvement by tumor mass and ECOG PS

Variable	p	OR	95% CI for OR
TP	0.026	1.91	1.08–3.39
Albumin	0.036	2.16	1.05–4.46
LDH	0.001	2.368	1.404–3.995
CRP	0.008	2.75	1.29–5.84
ESR	0.18	1.76	0.78–4.02
NLR	0.001	2.5	1.48–4.21
PLR	0.003	2.24	1.31–3.83
B-symptomatology	0.001	2.64	1.49–4.67
“Bulky” tumor mass	0.001	2.43	1.47–4.01
Mediastinal involvement	0.005	2.05	1.24–3.39
ECOG PS	0.015	1.99	1.14–3.5

Legend: *OR* odds ratio, *CI* confidence interval, *TP* total proteins, *LDH* lactate dehydrogenase, *CRP* C-reactive protein, *ESR* erythrocyte sedimentation rate, *NLR* neutrophil to lymphocyte ratio, *PLR* platelet to lymphocyte ratio, *ECOG PS* Eastern Oncology Cooperative Group Performance Status

**Fig. 1:**
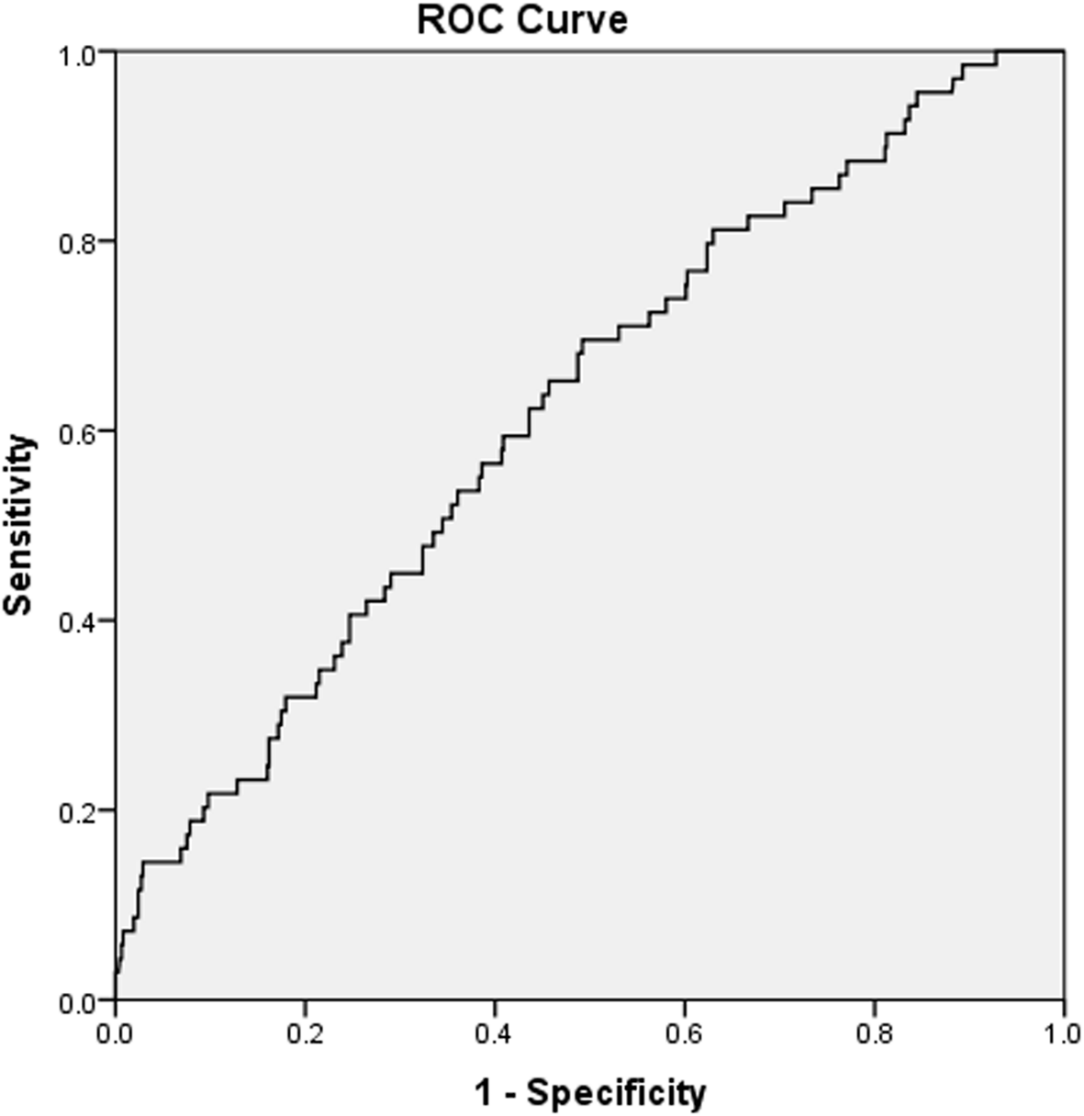
Receiver-operating characteristic curve of neutrophil to lymphocyte ratio

**Fig. 2:**
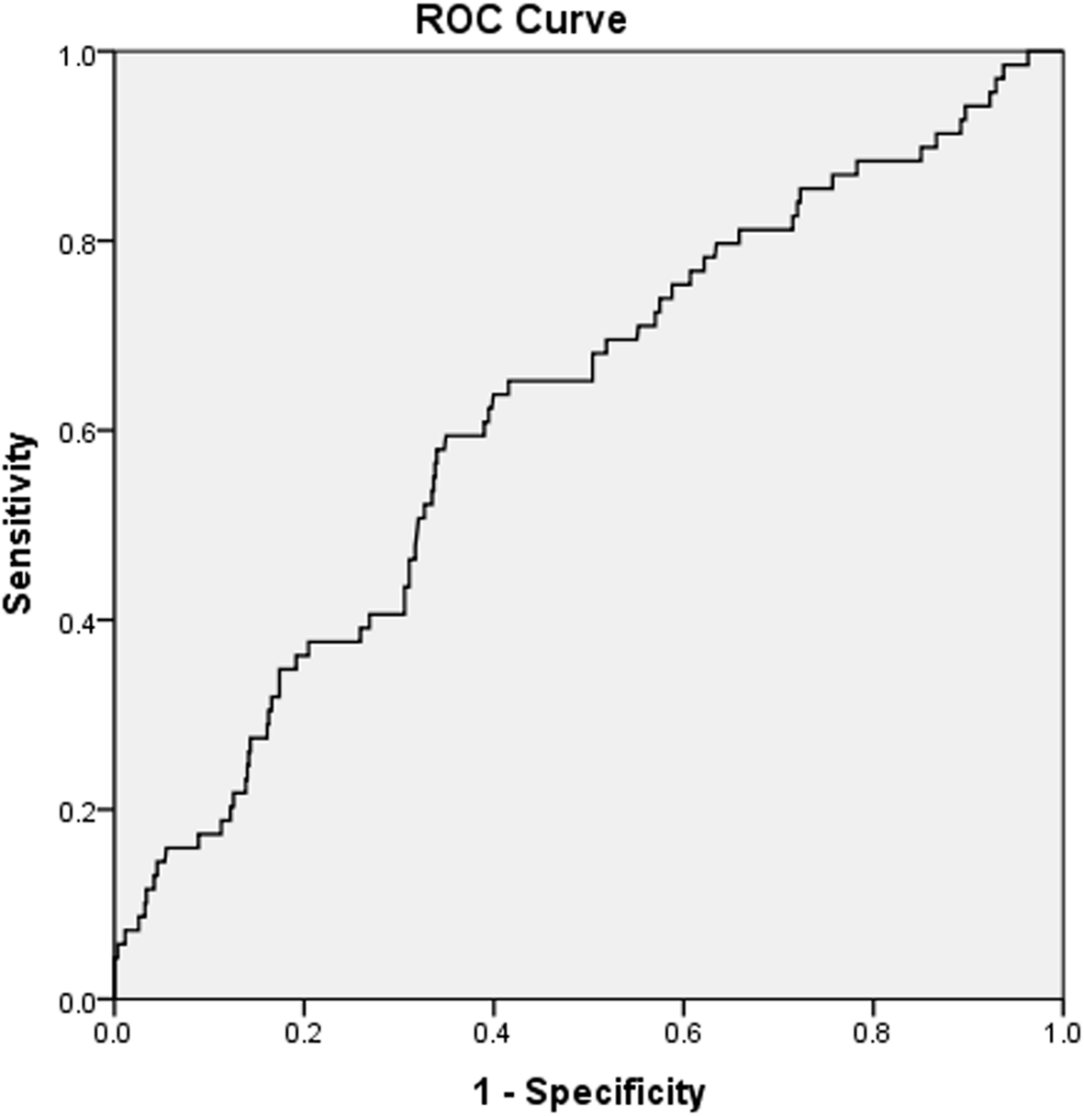
Receiver-operating characteristic curve of platelet to lymphocyte ratio

**Fig. 3:**
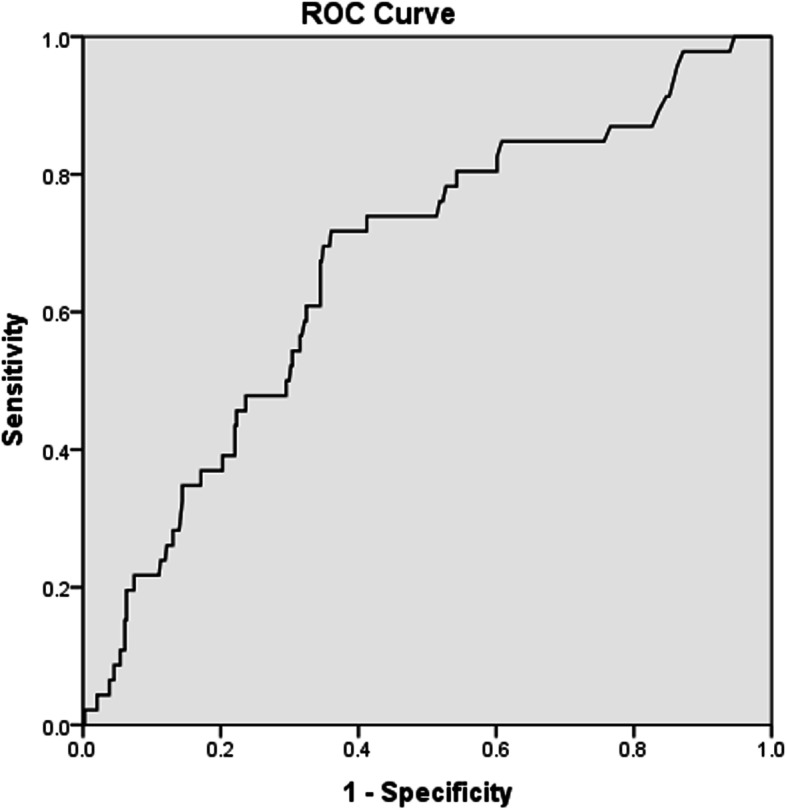
Receiver-operating characteristic curve of C-reactive protein

The ROC curve analysis demonstrated the following sensitivity and specificity values of NLR, PLR and CRP in predicting VTE (Sn = 65.2%, Sp = 57.1% for NLR; Sn = 69.6%, Sp = 49.7% for PLR; and Sn = 71.7%, Sp = 63.7% for CRP) (*p* = 0.001 for all) (Figs. 1, 2 and 3). A high NLR was defined as an NLR of 3 or higher, a high PLR as a PLR of 10 or higher, and a high CRP level as CRP > 20 mg/L. The Khorana score for previously defined cut-off values for high risk (≥3) were Sn = 11.6% and Sp = 88.0% in our study.

There was no difference in the use of thromboprophylaxis between the patients with lymphoma with and without VTE (13% vs. 18.4%, *p* = 0.268).

A poor therapeutic response to chemotherapy and immunotherapy was associated with the development of VTE (*p* = 0.011). Complete remission was less frequent in the patients with lymphoma who developed VTE than in those who did not develop VTE (36.9% vs. 53.6%, *p* = 0.011). The patients receiving intensive first-line or “salvage” chemotherapeutic regimens experienced a higher VTE rate than did those treated with standard first-line therapy regimens, such as R-CHOP, CHOP, and ABVD (18.2% vs 7.3%, *p* < 0.001).

## Discussion

In the study we aimed to evaluate the correlation between inflammatory markers and the risk of VTE development in a cohort of patients with lymphoma, and assess the relationship between VTE and treatment course in those patients. Our analysis found that the inflammatory markers correlated well with the risk for VTE development in patients with lymphoma, with NLR and CRP being the most accurate VTE predictive markers. Furthermore, we identified that an insufficient therapeutic response to (immuno) chemotherapy was a risk factor for VTE in the patients with lymphoma. Summarizing, immune dysregulation in lymphoma settings has a substantial impact on VTE occurrence.

In our study of patients with different types of lymphoma, the rate of VTE development was 9.8%. In a meta-analysis by Caruso et al., [21] which included 18,018 patients with lymphoma, the rate of VTE development was 6.4%. In that study, a higher rate of VTE development was observed in patients with NHL than in those with HL. In a study by Mahajan et al., [22] the cumulative 2-year incidences of acute VTE were 2.1, 4.8, and 4.5% in patients with low-grade, intermediate/aggressive, and high-grade lymphomas, respectively. Two studies [23, 24] focusing only on DLBCL found that the rate of VTE development was 11 and 11.1%, respectively. In a study examining the frequency of VTE in patients with cancer, Khorana et al. [25] observed that 4.8% of patients with NHL developed VTE, whereas 4.6% of those with HL developed VTE. In the study by Antic et al., [26] the rates of VTE development among patients with lymphoma were 5.3% in the derivation cohort and 5.8% in the validation cohort. In a recently published article [27] focusing on DLBCL and follicular lymphoma, the reported rate of VTE development was 13.4%. These observed variations in the VTE rate among patients with lymphoma are notable and may be caused by several factors, including focusing on distinctive types of lymphoma, study methodology (e.g., retrospective vs. prospective), and publication time (more recent studies have been dedicated to CAT). Our results are similar to those from studies focusing only on aggressive lymphoma, which is in accordance with the fact that more than half of our study population had aggressive lymphoma. In our cohort of patients with lymphoma with disease dissemination in the CNS, we have not observed any VTE. There are various causes that may have impacted the result. Just under 60% of patients with lymphoma with CNS disease were in satisfactory performance status at the time of therapy initiation. Moreover, during the last three years covered by the study, almost 70% of the patients were administered thromboprophylaxis, demonstrating higher adherence to the thromboprophylaxis guidelines in this specific subgroup of patients with lymphoma.

In our study, the NLR, PLR, ESR, CRP, and LDH were significantly higher in the patients with lymphoma with VTE than in those without VTE, whereas the TP and albumin were significantly lower in the patients with lymphoma with VTE than in those without VTE. The ROC curve analysis indicated acceptable specificity and sensitivity values of the NLR, PLR, and CRP in predicting VTE in the patients with lymphoma. In particular, the univariate regression analysis indicated that the NLR, PLR, TP, albumin, LDH, and CRP were prognostic factors for VTE development in the patients with lymphoma. However, the multivariate regression model demonstrated that only the NLR and CRP were independent prognostic factors for VTE development. Tumor-associated neutrophils have notable role in cancer microenvironment and serve as link between malignancy and inflammation, influencing cancer progression through several complex mechanisms. These mechanisms include mobilization of neutrophils from bone marrow towards tumor sites (mainly by CXCR2 axis), active participation in tumor microenvironment (release of reactive oxygen species and secretion of pro-tumor cytokines and chemokines) [28]. Likewise, platelets widely interact with tumor cells, whilst they have significant role in inflammation by releasing numerous inflammatory mediators, such as PF4 (CXCL4), P-selectin, and CD40L [29]. Both the NLR and PLR have been used as prognostic markers in a variety of pathological conditions, including sepsis, lupus erythematosus, and solid tumors [30]. Additionally, the NLR and PLR have been suggested as adverse prognostic markers in patients with DLBCL [31] and mantle cell lymphoma [32]. However, some studies have found conflicting results [33]. Regarding the association between the NLR and PLR with thrombotic events, some previous studies have shown the predictive power of the NLR and PLR for VTE development [16, 34]. In contrast, Artoni et al. [35] could not find an association between the NLR and PLR and an increased risk of VTE or cerebral vein thrombosis. To the best of our knowledge, there are no published studies on using the NLR and PLR to assess the risk of VTE in patients with lymphoma. Intermediate/high risk score of IPI in patients with DLBCL was significantly more frequent in the patients who developed VTE, comparing to those without VTE, which is in line with the recently published data [36].

An increasing number of studies aim to assess the relationship between inflammation and thrombosis, as well as the specific mechanisms underlying this relationship. However, the most validated mechanisms are yet to be discovered. The best studied mechanisms that have been shown to trigger thrombosis development or have been frequently observed in patients who develop thrombosis are increased levels of TNF-α, [37] hyperexpression of IL-6, [17] neutrophil extracellular traps, [38] soluble CD40L, [39] and microparticles (MPs) [40]. Kapoor et al. [41] significantly advanced our understanding of these processes by introducing a fourth element to Virchow’s triad-immune dysregulation, naming it the “tetrad of thrombosis.” They clearly stressed that there was a sufficient amount of evidence supporting the impact of immune dysregulation on the pathophysiology of thrombosis. A few studies identified higher CRP levels in patients with VTE (mainly DVT), [42] whereas the study by Antic et al. [43] published results similar to ours, showing the effect of a broad inflammatory and hemostatic biomarker spectrum (including D-dimer, Factor XIIIa, von Willebrand factor, TNF-α, protein S, β2Glycoprotein I, MPs, urokinase-like plasminogen activator, fibronectin, and plasminogen activator inhibitor type 1).

Similar to the results of previous studies, [21, 22, 44] we found that the patients with advanced stage disease more frequently developed VTE. However, this was not statistically significant. A “bulky” tumor mass, mediastinal involvement, and ECOG PS were identified as prognostic factors for VTE development in patients with lymphoma using the univariate analysis. A large mediastinal tumor mass is an important risk factor for the development of VTE, mainly due to the mechanical compression of blood vessels and consequent narrowing of the lumen [45, 46]. Performance status is included in newer VTE risk assessment models, underlining its importance in VTE development [26, 46]. Immobility has been recognized as a contributing factor for VTE development. It is of particular importance in patients with CNS lymphoma, as they have a strikingly high rate of VTE development (up to 59.5%) [47].

In our cohort, the patients with aggressive lymphoma had a higher rate of VTE development (11.8%) than did those with indolent lymphoma (5.3%) and HL (8.4%). Aggressive histology is predisposed to complicate the clinical course of lymphoma due to VTE occurrence [21, 22, 27, 44, 46, 48]. However, one large study by Sanfilippo et al. [49] concluded that the VTE risk for DLBCL was lowered after adjusting for additional risk factors. In general, aggressive lymphomas have higher proliferation rate that enables them to advance promptly and to obtain VTE risk factors more rapidly (“bulky” tumor mass, extranodal localizations, poor performance status), consequently increasing the risk for VTE development.

Complementary to our results, the predominant timing of VTE occurrence in patients with lymphoma was prior to or within three months from initiation of specific hematologic treatment [45, 50, 51]. These data draw attention to the role of thromboprophylaxis, which remains underused in cancer patients [26, 52]. Considering the absence of statistical significance for thromboprophylaxis between the patients with lymphoma with and without VTE, our data confirmed the underutilization of thromboprophylaxis. There are several reasons why thromboprophylaxis continues to be underused in patients with lymphoma: the lack of reliable and widely accepted usage of a VTE risk assessment model for this heterogeneous patient population, lack of prospective studies with risk stratification and randomization for thromboprophylaxis, [48] excessively diverse data throughout the literature concerning this topic, and overestimation of bleeding risk combined with anticoagulant therapy in cancer patients by clinicians. Further disease specific and appropriately designed clinical trials on thromboprophylaxis are required to achieve high quality evidence to ameliorate clinical guidelines.

Importantly, the patients with lymphoma who achieved unsatisfactory therapeutic responses were more susceptible to VTE development. This finding is in accordance with published data that confirmed the connection between aggressive lymphoma and advanced stage disease, resulting in shorter overall survival (OS) and a higher mortality rate [22, 44, 45, 53]. However, one study [54] did not observe an OS difference between the patients with lymphoma with and without VTE. The biology of aggressive lymphoma leads to aggravate clinical course. Moreover, immune dysregulation in patients with aggressive lymphoma subtypes is probably impaired to a greater extent, which contributes to the risk for VTE occurrence.

In our study, the patients receiving intensive first-line or “salvage” chemotherapeutic regimens experienced a higher rate of VTE development than those treated with standard first-line therapy regimens (R-CHOP, CHOP, and ABVD). Chemotherapy itself is known to be a risk factor for VTE development [3, 46]. The incidence of VTE was higher in the patients with lymphoma treated with dose-intense regimens [55]. Furthermore, anthracycline drugs were associated with an increased risk of VTE [27, 49]. Intensive first-line therapeutic regimens are used to treat more aggressive lymphoma subtypes, and both intensive regimens and aggressive subtypes are potential risk factors for VTE development. Relapsing lymphomas are inclined to follow a more aggressive clinical course, primarily due to the disease biology and development of resistant features. Consequently, those patients are treated with more intensive, so-called “salvage,” chemotherapeutic regimens. These patients frequently have other VTE risk factors, such as poor performance status and advanced stage disease, which significantly increase the risk of VTE development.

Corresponding to several previous findings, [56, 57] the Khorana score has not demonstrated satisfactory thrombotic prediction performance in the patients with lymphoma. Therefore, the paradigm of shifting towards disease specific risk assessment models (RAMs) increasingly prevail in recent years in the lymphoma field as well.

Our study has several limitations. The main limitation is the heterogeneity of the study population, which possibly might have affected the results and subsequent conclusions. The impact of VTE onto survival rates of lymphoma patients was out of scope in this study. Perhaps, that would further contribute to the assessment of actual clinical impact of VTE in patients with lymphoma.

## Conclusions

In conclusion, immune activation represents a distinctive feature of lymphomas, especially aggressive lymphomas. Dysregulation of inflammation plays an important role in VTE development in patients with lymphoma. The findings of this study demonstrated that easily and widely accessible simple parameters that reflect the level of inflammation have the ability to identify patients with lymphoma at risk for VTE who may be candidates for thromboprophylaxis. In addition, the possible use of anti-inflammatory drugs in this specific group of patients would extend the tools for VTE prophylaxis. Further studies are required to better understand VTE in lymphoma settings and the utilization of these inflammatory markers in VTE risk assessment.

## Supplementary Information

**Additional file 1**

## Data Availability

The datasets used and analysed during the current study are available from the corresponding author on reasonable request.

## Abbreviations

VTE: Venous thromboembolism
CAT: Cancer-associated thrombosis
WBC: White blood cell
CRP: C-reactive protein
NLR: Neutrophil-to-lymphocyte ratio
PLR: Platelet-to-lymphocyte ratio
NHL: Non-Hodgkin lymphoma
HL: Hodgkin lymphoma
CLL: Chronic lymphocytic leukemia
UCCS: University Clinical Center of Serbia
CNS: Central nervous system
CBC: Complete blood count
ESR: Erythrocyte sedimentation rate
LDH: Lactate dehydrogenase
TP: Total protein
ROC: Receiver operating characteristic
OR: Odds ratio
CI: Confidence intervals
ECOG PS: Eastern Cooperative Oncology Group Performance Status
DVT: Deep vein thrombosis
DLBCL: Diffuse large B-cell lymphoma
TNF-α: Tumor necrosis factor-alpha
MP: Microparticles
OS: Overall survival
