# Supplementary figures and images for: Immune activation and inflammatory biomarkers as predictors of venous thromboembolism in lymphoma patients
Source: Thromb J. 2022 Apr 19;20:20. doi: 10.1186/s12959-022-00381-3 (PMC9016935; doi:10.1186/s12959-022-00381-3)

TUMOR MICROENVIRONMENT

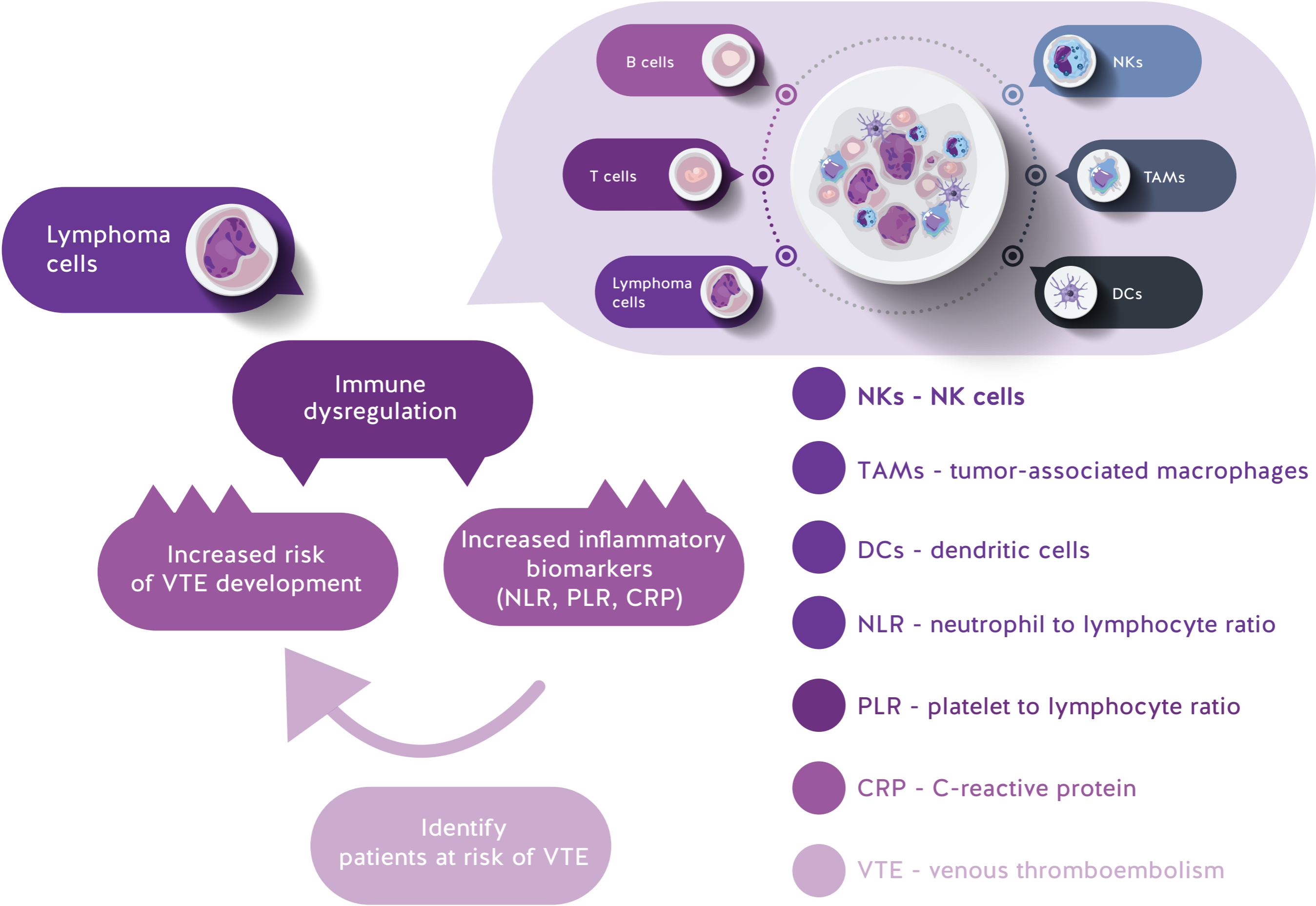

Supplement: Supplementary file 1 — Additional file 1 [file 12959_2022_381_MOESM1_ESM.pdf]
